# Silicon induces hormetic dose-response effects on growth and concentrations of chlorophylls, amino acids and sugars in pepper plants during the early developmental stage

**DOI:** 10.7717/peerj.9224

**Published:** 2020-06-09

**Authors:** Libia Iris Trejo-Téllez, Atonaltzin García-Jiménez, Hugo Fernando Escobar-Sepúlveda, Sara Monzerrat Ramírez-Olvera, Jericó Jabín Bello-Bello, Fernando Carlos Gómez-Merino

**Affiliations:** 1 Department of Soil Science. Laboratory of Plant Nutrition, College of Postgraduates in Agricultural Sciences Campus Montecillo, Texcoco, State of Mexico, Mexico; 2 Department of Plant Physiology, College of Postgraduates in Agricultural Sciences Campus Montecillo, Texcoco, State of Mexico, Mexico; 3 Institute of Biological Sciences, University of Talca, Talca, Maule, Chile; 4 Department of Biotechnology, CONACYT-College of Postgraduates in Agricultural Sciences Campus Córdoba, Amatlán de los Reyes, Veracruz, Mexico

**Keywords:** Solanaceae, *Capsicum annuum*, Beneficial elements, Orthosilicic acid, Hormesis, Seedlings

## Abstract

**Background:**

Silicon (Si) is a beneficial element that has been proven to influence plant responses including growth, development and metabolism in a hormetic manner.

**Methods:**

In the present study, we evaluated the effect of Si on the growth and concentrations of chlorophylls, total amino acids, and total sugars of pepper plants (*Capsicum annuum* L.) during the early developmental stage in a hydroponic system under conventional (unstressed) conditions. We tested four Si concentrations (applied as calcium silicate): 0, 60, 125 and 250 mg L^−1^, and growth variables were measured 7, 14, 21 and 28 days after treatment (dat), while biochemical variables were recorded at the end of the experiment, 28 dat.

**Results:**

The application of 125 mg L^−1^ Si improved leaf area, fresh and dry biomass weight in leaves and stems, total soluble sugars, and concentrations of chlorophylls *a* and *b* in both leaves and stems. The amino acids concentration in leaves and roots, as well as the stem diameter were the highest in plants treated with 60 mg L^−1^ Si. Nevertheless, Si applications reduced root length, stem diameter and total free amino acids in leaves and stems, especially when applied at the highest concentration (i.e., 250 mg L^−1^ Si).

**Conclusion:**

The application of Si has positive effects on pepper plants during the early developmental stage, including stimulation of growth, as well as increased concentrations of chlorophylls, total free amino acids and total soluble sugars. In general, most benefits from Si applications were observed in the range of 60–125 mg L^−1^ Si, while some negative effects were observed at the highest concentration applied (i.e., 250 mg L^−1^ Si). Therefore, pepper is a good candidate crop to benefit from Si application during the early developmental stage under unstressed conditions.

## Introduction

Silicon (Si), only after oxygen (O), is the second most abundant element in the Earth’s crust, covering up to 32% of the lithosphere (Savant et al., 1999; Manivannan et al., 2016). In nature, Si is found as silicates and Si minerals, combined with O or elements like aluminum (Al), manganese (Mg), calcium (Ca), sodium (Na), iron (Fe) and potassium (K), mainly, in over 95% of earthly rocks, meteorites, water and the atmosphere (Artyszak, 2018). In plants, Si can only be absorbed as monosilicic acid (Si(OH)_4_), and it is transported and mainly deposited in the cell apoplast. Generally, Si concentrations in plants fluctuate between 0.1% and 10% of the total dry matter (Epstein, 1994), which primarily depends on the plant genotypes and secondly on soil properties as a source of Si (Coskun et al., 2019). It is worth mentioning that seven out of the 10 most produced crops in the world (ranked by quantity) are Si accumulators (Guntzer, Keller & Meunier, 2012) and most of them positively respond to Si applications (Gómez-Merino & Trejo-Téllez, 2018). These crops include rice (*Oryza sativa* L.), wheat (*Triticum aestivum* L.), barley (*Hordeum vulgare* L.), sugarcane (*Saccharum* spp. L.), soybean [*Glycine max* (L.) Merr.] and sugarbeet (*Beta vulgaris* L. subsp. *vulgari*s) (Guntzer, Keller & Meunier, 2012; Elsokkary, 2018; Artyszak, Gozdowski & Kucińska, 2019).

In Si accumulator species, Si absorption can cause beneficial effects (Guntzer, Keller & Meunier, 2012). When plants are grown under conventional environments (i.e., not subject to stress), Si probably makes plants more efficient in responding to environmental cues by activating different metabolic processes (Luyckx et al., 2017a) with crucial cascading effects on plant structure and function (Guntzer, Keller & Meunier, 2012; Coskun et al., 2019). Si has biostimulant effects on plants (Epstein, 2009; Gómez-Merino & Trejo-Téllez, 2018) by modifying physiological processes in a way that provides benefits to growth, development or stress responses (Savvas & Ntatsi, 2015). Monocotyledons and especially species belonging to the family Poaceae such as rice and sugarcane respond positively to Si supply (Epstein, 1999; Ma et al., 2007), but many other dicotyledons including species of the families Fabaceae and Cucurbitaceae respond to Si applications too, especially when plants are exposed to biotic or abiotic stress (Ma, 2004; Fauteux et al., 2005). Therefore, Si has been regarded as a “quasi-essential” element for higher plants, in the sense that Si fertilization can enhance plant growth and yield, while Si starvation may hamper normal metabolisms and cause physical disorders (Rafi, Epstein & Falk, 1997; Ma & Yamaji, 2008).

Importantly, Si may differentially affect plant growth and metabolism depending on the source and concentration applied, which may be attributed to chemically induced hormesis. In nature, widespread and frequent hormetic-like biphasic dose–responses occur across the broad spectrum of life including plants (Calabrese et al., 2019; Agathokleous & Calabrese, 2020). Hormesis is a biphasic dose–response relationship with low doses inducing stimulatory effects by activating adaptive mechanisms that enhance resilience, while higher doses may induce inhibitory responses that at even higher doses often become toxic (Agathokleous & Calabrese, 2019a, 2019b; Agathokleous & Calabrese, 2020). In banana (*Musa* spp. L.), the application of 200 mg Si per week resulted in a stimulatory effect leading to the beneficial growth attributes, whereas treatments with 500 and 1,000 mg Si per week triggered inhibitory responses, resulting in detrimental effects evidenced by stunting and discoloration of the leaf edges (Mburu et al., 2016). Negative effects such as stunting, deformed flowers and delay in flowering were also observed in sunflowers at high concentrations of Si, thus suggesting Si application can vary from beneficial to detrimental depending on the source and concentration used (Kamenidou, Cavins & Marek, 2008; Kamenidou, Cavins & Marek, 2010). Hence, we hypothesized that Si can trigger beneficial effects on growth and metabolism of pepper seedlings under conventional conditions (i.e., unstressed), and that this effect would depend on the concentrations of Si tested in a hormetic manner. Concomitantly, herewith we aimed at evaluating the effect of increasing levels of Si applied through the nutrient solution in a hydroponic system on the performance of pepper seedlings, in order to gain a better insight into the potential hormetic role of this element on plants grown under conventional environments (i.e., not subject to stress) during the early developmental stage. We evaluated different parameters related to growth, biomass accumulation, and concentrations of chlorophylls, sugars and amino acids in plant tissues (i.e., roots, stems and leaves) in response to the application of four levels of Si (i.e., 0, 60, 125 and 250 mg L^−1^ Si) supplied as calcium silicate (CaSiO_3_) in the nutrient solution.

## Materials and Methods

### Plant material and growing conditions

The experiment was carried out in a greenhouse at the College of Postgraduates Campus Montecillo, Mexico (98° 91′ W, 19° 45′ N, 2,224 masl). Pepper (*Capsicum annuum* L.) cv. Mysterio seeds (Harris-Moran Seeds, Querétaro, Querétaro, Mexico) as previosly described (García-Jiménez et al., 2018). Thirty-d-old seedlings were transplanted into 35 L plastic containers supplied with the Steiner nutrient solution (Steiner, 1984) (at 20% or the original strength) supplemented with micronutrients.

Seven days after transplanting, the nutrient solution was completely renewed and the treatments to be tested (different Si concentrations) were added. The treatments consisted of 60, 125 and 250 mg L^−1^ Si, and the control consisted of the Steiner nutrient solution without Si. Si was supplied as CaSiO_3_ (*purum* grade, with ≥87% SiO_2_ basis, 12–22% Ca (as CaO) basis) (Sigma–Aldrich, St. Louis, MO, USA). The Steiner nutrient solution supplied at 20% of the original strength, is sufficient, but not excessive, to grow pepper seedlings at this developmental stage (García-Jiménez et al., 2017). In order to guarantee the availability of all essential elements, the nutrient solution was completely replaced every seven days. Furthermore, the nutrient solution was aerated every 2 h for 15 min with an air pump (Hagen, Elite 802; Manfield, MA, USA), adjusting the pH to 5.5 with concentrated 1 N NaOH or H_2_SO_4_ (Sigma–Aldrich, St. Louis, MO, USA).

The experimental unit was represented by a single pepper plant, and each treatment had 12 replicates, which were distributed in a completely randomized experimental design. The experiment was conducted under greenhouse conditions as described elsewhere (García-Jiménez et al., 2018). The greenhouse was illuminated with natural sunlight. It is worth mentioning that the light requirements of plants are 100–300 μmol m^−2^ s^−1^ of photosynthetic photon flux density (PPFD) for leafy vegetables, 200–600 μmol m^−2^ s^−1^ for fruiting vegetables, and 50–200 μmol m^−2^ s^−1^ for ornamental plants (Wada, Fukuda & Ogura, 2019). Since our plants were in the seedling stage, we used 300 μmol m^−2^ s^−1^ PPFD.

### Physiological and biochemical measurements

#### Evaluation of plant growth and development

Seven, 14, 21 and 28 days after treatment application (dat) we measured the variables plant height and root length using a 30 cm stainless steel ruller as previously described (García-Jiménez et al., 2018). The number of leaves and flower buds, stem diameter, root volume, leaf area, weight of fresh and dry root, stem, leaf and flower biomass were evaluated 28 dat as described elsweher (García-Jiménez et al., 2017, 2018).

#### Concentrations of chlorophylls *a*, *b* and total chlorophylls in leaves and stems

Chlorophyll concentrations were determined following the Harborne method (Harborne, 1973). Accordingly, after a triple ethanol extraction, samples obtained were incubated in a water bath, centrifuged and read in a 6715 UV/Vis spectrophotometer (Jenway, Staffordshire, UK) at 645 and 665 nm. From the readings we could calculate the concentrations of chlorophylls *a* and *b*. Total chlorophyl concentration was the sum of chlorophyl *a* and *b*, and we also determined the corresponding ratios of chlorophylls *a*/*b*.

#### Concentrations of total free amino acids in leaves, stems and roots

The ninhydrin method (Moore & Stein, 1954; modified by Sun et al. (2006)) was used to determine the concentrations of total free amino acids in plant tissues. Accordingly, 500 μL of the triple ethanol extraction was taken and mixed with 500 μL of the Na citrate (16 mM)-ascorbic acid (34 mM) buffer solution at 0.2% (w/v), pH of 5.2 and 1,000 μL ninhydrin (1%; w/v) in 70% ethanol (v/v) were also added. After incubation 95 °C (20 min) and having left them to cooled down at room temperature, samples were read in the 6,715 UV/Vis spectrophotometer at 570 nm, using leucine to obtain the calibration curve. For each treatment, four replicates were prepared, with two technical replicates each.

#### Concentrations of total soluble sugars in leaves, stems and roots

The concentration of total soluble sugars was estimated with the anthrone method (Brummer & Cui, 2005; based on Southgate, 1976). After extraction with 80% ethanol at 125 °C, samples were filtered and measured to a volume of 20 mL, from which 500 μL were taken and mixed with 500 μL of 80% ethanol. Subsequently, five mL of cooled anthrone (Meyer, Querétaro, Querétaro, Mexico) dissolved in concentrated sulfuric acid (Merck KGaA, Darmstadt, Germany) were added to the samples and placed on ice. Samples were then transferred to a water bath at 95 °C for 15 min, and then placed back on ice to cool down. A standard curve was done with glucose (Sigma–Aldrich, St. Louis, MO, USA) and the samples were read at 620 nm in the 6,715 UV/Vis spectrophotometer. For each treatment, four replicates were prepared, with two technical replicates each.

### Statistical analysis

The assumptions of normality and homogeneity of variances of our experimental data were verified through the Shapiro–Wilk and Bartlett tests (*P* ≤ 0.05), respectively. When either of these assumptions was not fulfilled, a logarithmic transformation was done, although the data are shown without transforming. Subsequently, a one-way analysis of variance was carried out. When there were statistical differences, mean separation was done through the Duncan method with α = 0.05. The SAS 9.0 software (SAS Institute, Cary, NC, USA) was used for all analyses.

## Results

### Plant growth and development

**Figure 1:**
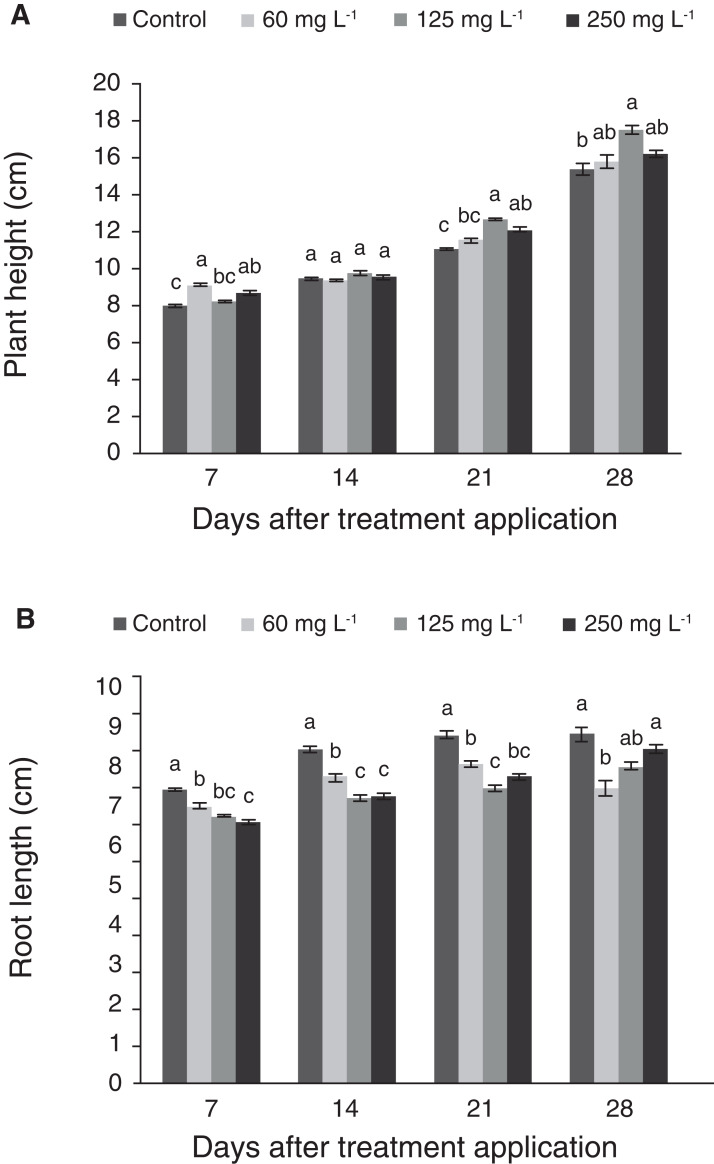
Plant height (A) and root length (B) of pepper plants (*Capsicum annuum* L.) grown in nutrient solutions containing different concentrations of Si under unstressed conditions. Error bars indicate standard deviation. Columns with different letters are statistically different (*P* ≤ 0.05).

**Figure 2:**
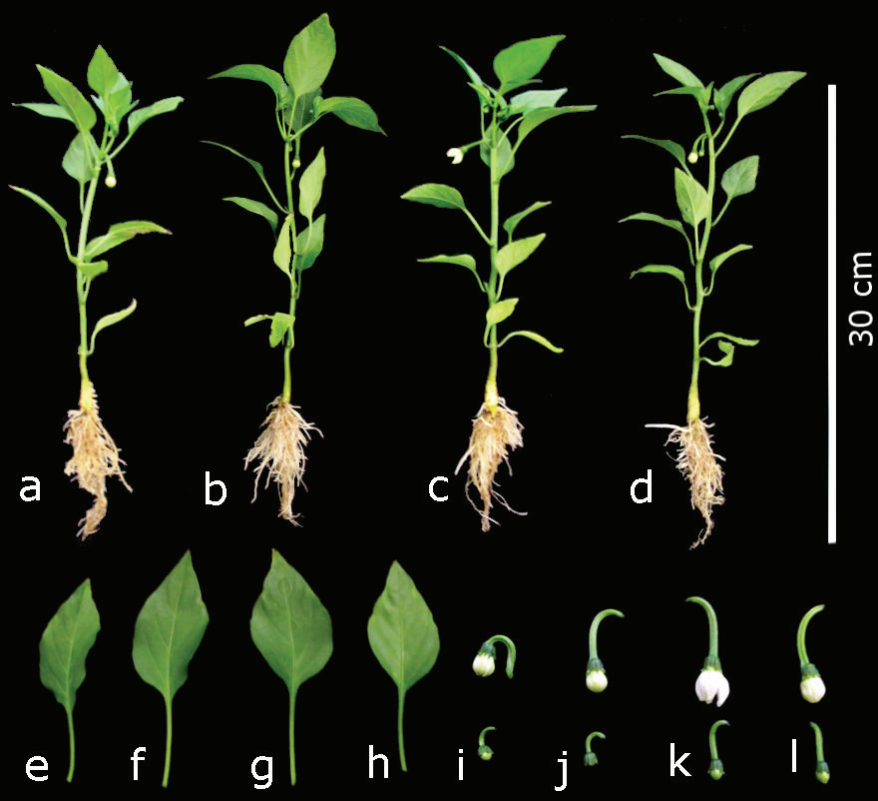
Development of pepper plants (*Capsicum anunum* L.) grown in nutrient solutions containing different concentrations of Si under unstressed conditions 28 dat. Control: (A), (E) and (I); 60 mg L^−1^ Si: (B), (F) and (J); 125 mg L^−1^ Si: (C), (G) and (K); 250 mg L^−1^ Si: (D), (H) and (L).

Treatments (0, 60, 125 and 250 mg L^−1^ Si) were applied to 37-d-old plants in hydroponics, and variables were measured weekly during 4 weeks. Seven days after the application of the treatments (dat), plant height was significantly greater with the application of 60 and 250 mg L^−1^ Si in comparison to the control, while at 14 days there were no significant differences among treatments. At 21 d, plant height increased significantly in the treatments with higher Si concentrations (125 and 250 mg L^−1^), while there were no significant differences between the control and the treatment with 60 mg L^−1^ Si. In the last evaluation, the plants treated with 125 mg L^−1^ Si had the greatest height, with no significant differences in comparison to the other two treatments with Si (Fig. 1A). Although the control recorded the lowest height, this mean was not statistically different to those observed in plants treated with 60 and 250 mg L^−1^ Si. In contrast to plant height, root length in control plants was greater than all the other treatments at 7, 14 and 21 dat. At 28 d, plants treated with 125 and 250 mg L^−1^ Si had longer roots than those treated with 60 mg L^−1^ Si, but this mean was not statistically different as compared to the control (Fig. 1B). Figure 2 displays how plants were phenotypically affected by Si treatments.

Silicon also stimulated reproductive responses. Indeed, the development of flowering in plants treated with 125 mg L^−1^ Si was faster in comparison to the rest of the treatments 28 dat. Though there were no flowers yet in plants treated with 60 and 250 mg L^−1^ Si, in those two treatments flower buds were larger than those of the control (Figs. 2I, 2J, 2K and 2L).

**Figure 3:**
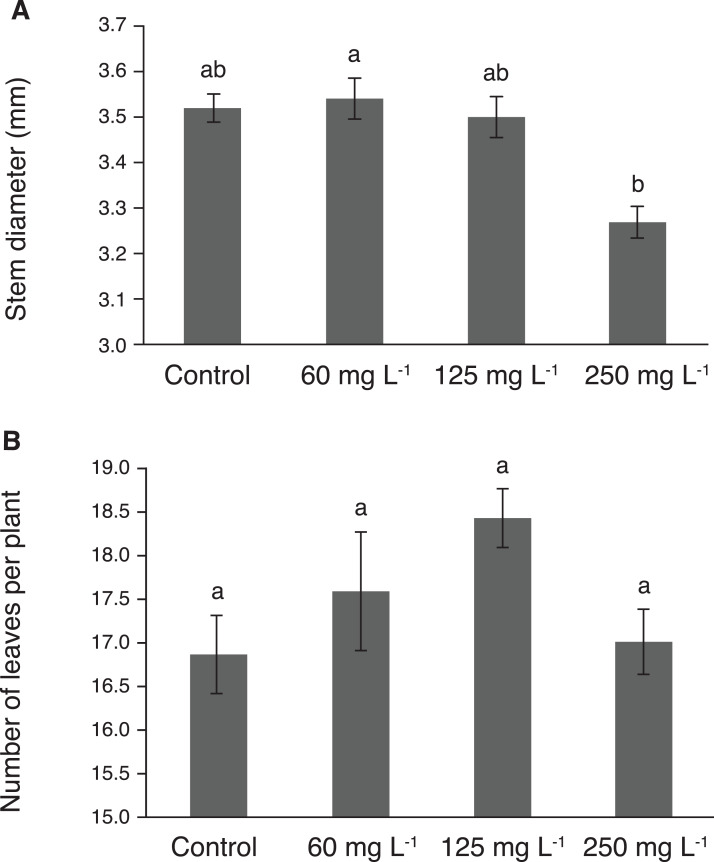
Stem diameter (A) and number of leaves (B) in pepper plants (*Capsicum annuum* L.) grown in nutrient solutions containing different concentrations of Si under unstressed conditions. Error bars indicate standard deviation. Columns with different letters are statistically different (*P* ≤ 0.05).

Stem diameter reached its highest value in plants treated with 60 mg L^−1^, though this value was statistically similar to those observed in plants exposed to 125 mg L^−1^ and the control (Fig. 3A). Importantly, the lowest stem diameter was observed in plants exposed to 250 mg L^−1^ Si. The number of leaves per plant showed no significant effect due to the treatments tested (Fig. 3B). However, the treatment with 125 mg L^−1^ had 9% more leaves than the control.

There were no significant differences between the treatments with Si and the control with regard to root volume, number of flower buds per plant, and the weight of fresh and dry biomass of flowers (Table 1). With the application of 125 mg L^−1^ Si, plants developed larger leaves than with the other treatments, including the control (Figs. 2E, 2F, 2G and 2H), which consequently resulted in plants treated with this Si concentration having a larger leaf area. Control plants and those exposed to 60 mg L^−1^ Si displayed similar leaf area, which was lower than the means observed in plants treated with 125 mg L^−1^ Si (Table 1).

**Table 1:** Root volume, leaf area, number of flower buds per plant, and weight of fresh and dry flower biomass in pepper (*Capsicim annuum* L.) grown in nutrient solutions containing different concentrations of Si under unstressed conditions, at 28 dat.

Si treatment (mg L^−1^)	Root volume (mL)	Leaf area (cm^2^)	Flower buds	Weight of fresh flower biomass (mg)	Weight of dry flower biomass (mg)
Control	2.20 ± 0.06 a	50.85 ± 1.19 b	3.50 ± 0.11 a	131.25 ± 8.41 a	17.73 ± 1.67 a
60	1.80 ± 0.10 a	50.80 ± 1.32 b	4.80 ± 0.34 a	210.25 ± 23.39 a	36.90 ± 5.65 a
125	2.00 ± 0.06 a	66.03 ± 3.61 a	4.50 ± 0.25 a	183.25 ± 5.31 a	32.43 ± 3.51 a
250	1.75 ± 0.06 a	54.73 ± 1.99 ab	4.10 ± 0.22 a	173.25 ± 8.38 a	32.65 ± 2.90 a
*P*	0.1792	0.1046	0.2991	0.2645	0.3305

**Note:**

Values are means ± standard deviation (SD) from at least five individual plants. Different letters in each column indicate significant differences among treatments for each variable analyzed (Duncan, *P* ≤ 0.05).

**Figure 4:**
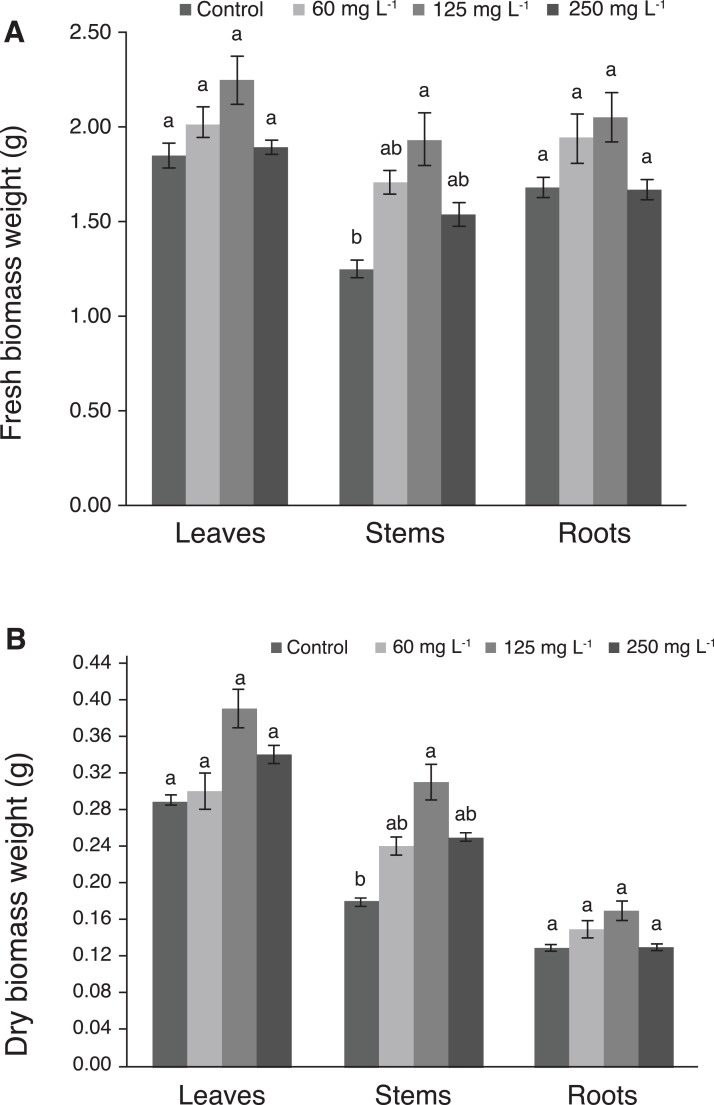
Weight of fresh (A) and dry (B) biomass of leaves, stems, and roots of pepper plants (*Capsicum annuum* L.) grown in nutrient solutions containing different concentrations of Si under unstressed conditions 28 dat. Error bars indicate standard deviation. Columns with different letters are statistically different (*P* ≤ 0.05).

Fresh and dry biomass weights of leaves and roots were not significantly different among treatments. Interestingly, plants treated with 125 mg L^−1^ Si had the greatest weight of fresh and dry stem biomass, which was statistically different from the control (Figs. 4A and 4B).

### Chlorophylls concentration in leaves and stem

The highest concentration of chlorophyl *a* found in leaves and stems was recorded in plants treated with 125 mg L^−1^ Si, while the application of 250 mg L^−1^ Si reduced the concentration of this molecule in both tissues analyzed to levels similar to the control (Table 2). The concentration of chlorophyl *b* in leaves was not significantly different among 60, 125 mg L^−1^ Si, and the control, while with the application of 250 mg L^−1^ Si the lowest chlorophyl *b* concentration was observed. In stems, the highest concentration of chlorophyl *b* was observed in plants treated with 125 mg L^−1^ Si, while the application of 250 mg L^−1^ Si decreased the value of this variable to an even lower level than the control. Total chlorophyl in leaves was the highest in plants receiving 125 mg L^−1^ Si, while the lowest concentration was obtained with 250 mg L^−1^ Si. Similarly, plants treated with 125 mg L^−1^ Si displayed the highest means for total chlorophylls in stems, while the lowest values were observed in plants exposed to 60 and 125 mg L^−1^ Si; plants receiving 250 mg L^−1^ and the control showed intermediate concentrations of total chlorophylls, which were statistically similar to each other. In both leaves and stems, the chlorophyl *a*/*b* ratio was the highest in plants treated with 250 mg L^−1^; it was observed that as the Si concentration decreased, so did the chlorophyl *a*/*b* ratio.

**Table 2:** Chlorophyll concentration (mg g^−1^ FBW) in leaves and stems of pepper (*Capsicum annuum* L.) grown in nutrient solutions containing different concentrations of Si under unstressed conditions, at 28 dat.

Si treatment (mg L^−1^)	Chlorophyll concentrations (mg g^−1^ FBW)							
	Chlorophyll *a*		Chlorophyll *b*		Total Chlorophylls		Chlorophyll *a/b* ratio	
	Leaf	Stem	Leaf	Stem	Leaf	Stem	Leaf	Stem
Control	1,513.73 ± 8.5c	348.95 ± 5.2b	291.82 ± 6.2a	112.65 ± 1.7b	1,805.55 ± 12.9c	461.60 ± 4.3b	5.21 ± 0.09c	3.10 ± 0.05b
60	1,611.05 ± 3.2b	318.29 ± 5.8c	284.21 ± 5.5a	100.09 ± 3.9bc	1,895.25 ± 8.2b	418.38 ± 4.0c	5.69 ± 0.2bc	3.24 ± 0.13b
125	1,691.16 ± 10.4a	429.15 ± 4.7a	284.05 ± 7.7a	144.20 ± 1.3a	1,975.21 ± 12.4a	573.36 ± 7.5a	6.0 ± 0.1b	2.97 ± 0.02b
250	1,478.39 ± 7.7c	371.84 ± 4.2b	171.79 ± 2.6b	85.09 ± 2.1c	1,650.17 ± 10.1d	456.93 ± 2.3b	8.62 ± 0.1a	4.40 ± 0.11a
*P* value	<0.0001	<0.0001	<0.0001	<0.0001	<0.0001	<0.0001	<0.0001	0.0004

**Note:**

Values are means ± standard deviation (SD) from at least five individual plants. Different letters in each row indicate significant differences among treatments for each variable analyzed (Duncan, *P* ≤ 0.05). FBW, fresh biomass weight.

### Concentration of total free amino acids and total soluble sugars

**Figure 5:**
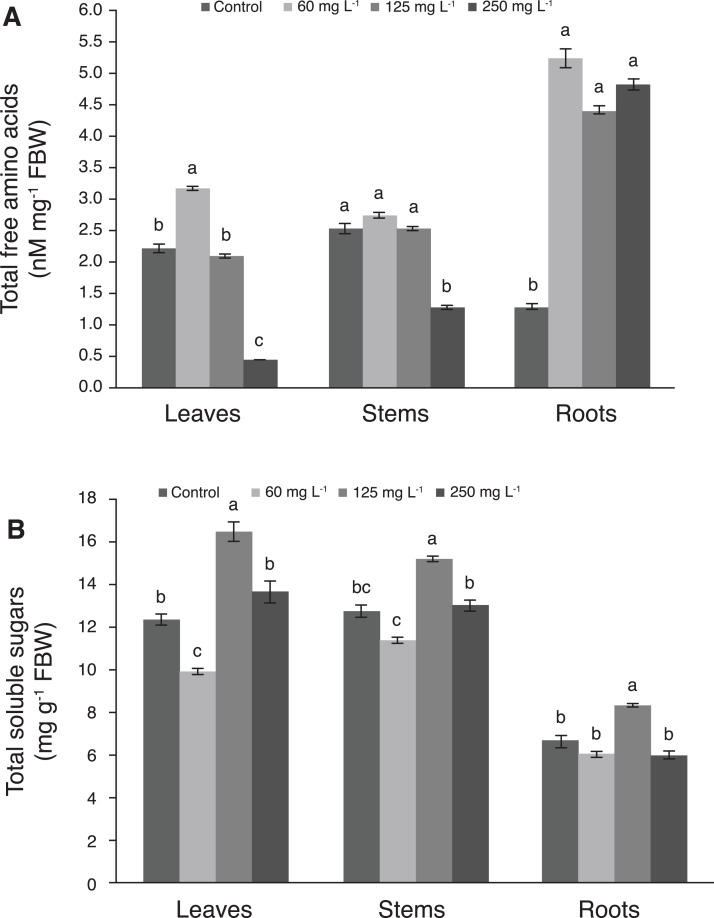
Concentration of total free amino acids (A) and total soluble sugars (B) in leaves, stems, and roots of pepper plants (*Capsicum annuum* L.) grown in nutrient solutions containing different concentrations of Si under unstressed conditions 28 dat. Error bars indicate standard deviation. Columns with different letters are statistically different (*P* ≤ 0.05). FBW, fresh biomass weight.

The concentration of amino acids in leaves was 42.9% greater with 60 mg L^−1^ Si than in the control. Moreover, as the Si concentration increased, the amino acids concentration was decreased in this tissue, since the lowest values were recorded with 250 mg L^−1^ Si. In stems, the concentration of amino acids decreased drastically with the application of 250 mg L^−1^ Si, while plants treated with 60 and 125 mg L^−1^ Si were statistically similar to the control. In roots, control plants had the lowest concentration of amino acids, while Si stimulated the concentrations of these biomolecule in this tissue in all levels evaluated (Fig. 5A). Comparing amino acids concentrations among plant tissues, the highest values were observed in roots.

The concentration of total soluble sugars in leaves of plants treated with 125 mg L^−1^ Si was 33.1% higher than in those of the control, while the 250 mg L^−1^ Si treatment and the control were not statistically different from each other. Likewise, in stems, the concentration of sugars in plants treated with 125 mg L^−1^ Si was 19.5% higher than the control, while 60 and 250 mg L^−1^ Si were not significantly different from the control. In roots, plants treated with 125 mg L^−1^ Si recorded the highest sugar concentration, which was similar to the control (Fig. 5B).

## Discussion

### Si stimulates growth of pepper plants in a hormetic manner

In horticulture, the seedling stage is important within the crop cycle because it affects growth and development, earliness, total yield and fruit number per plant (Demir et al., 2010). Seedling quality can be assessed by measuring morphological, physiological and biochemical traits (Aragão et al., 2015; Ritchie, 1984). Among the morphological attributes to be considered are height, stem diameter, root biomass, and stem biomass, to cite some, while biochemical traits involve soluble sugars, amino acids and polyamines, among others (Aragão et al., 2015; Haase, 2008; Larson et al., 2015; Pessoa et al., 2019; De Souza Vidigal et al., 2011). High quality seedlings have a higher survival rate and faster growth in the field than poor quality ones, which has both agronomic and economic benefits. Particularly, the initial growth phase of sweet pepper seedling represents a decisive stage for the commercial production of both seedlings and fruits. The rate of seedling emergence, their uniformity and initial growth determine seedling quality, affecting the overall economic efficiency of the production system (De Grazia, Tittonell & Chiesa, 2004) and beneficial elements such as Si may boost its performance. Herein we aimed to evaluate the effect of adding different concentrations of Si in the nutrient solution on quality attributes of pepper seedlings.

In previous studies, we have reported the effect of a number of Si concentrations on growth, development, and nutrient concentration of heliconia (*Heliconia psittacorum* L.f. × *H*. *spathocircinata* Aristeguieta cv. Golden Torch Adrian) (Cuacua-Temiz et al., 2017) and sugarcane (*Saccharum* spp. L.) (Sentíes-Herrera et al., 2018). Moreover, we performed an in-depth analysis of the literature on Si dosage resulting in beneficial effects to diverse plant species, which we published as a part of a book chapter (Gómez-Merino & Trejo-Téllez, 2018). As a chemical element causing hormetic responses in plants, Si can not only benefit the plant, but it can also result in negative effects. Indeed, excess levels of Si can potentially compete with uptake of other nutrients and affect plant metabolism. In sunflower (*Helianthus annuus* L. cv. “Ring of Fire”), growth abnormalities were observed when concentrations of 100 and 200 mg L^−1^ Si were supplied as KSiO_3_ substrate drenches (Kamenidou, Cavins & Marek, 2008). In these treatments, plants appeared stunted with deformed flowers and were delayed in flowering. In gerbera (*Gerbera* sp. L. hybrid “Acapella”), foliar sprays of 150 mg Si L^−1^ supplemented as NaSiO_3_ resulted in stem shortening and deformation of flowers (Kamenidou, Cavins & Marek, 2010). In general, plants that are considered “non-accumulators” of Si are more sensitive to excess Si compared to those that are “accumulators” (Bloodnick, 2018). Considering these reports and our experimental data, we decided to perform further analyses by comparing the effect of applying 0 (control), 60, 125 and 250 mg L^−1^ Si (supplied as CaSiO_3_) on growth, concentrations of some vital biomolecules crucial for seedling development and nutrients in pepper plants at the early stage of development under unstressed conditions.

Our experimental results show a number of beneficial effects of Si on pepper plant growth and metabolism under conventional environmental conditions (i.e., in the absence of stress) when applied at low to medium levels, whereas high levels resulted in detrimental effects to the plants. Plant height, stem diameter, leaf area, fresh and dry biomass weight, as well as the concentraions of chlorophylls, total amino acids and total soluble sugars were enhanced in pepper plants treated with Si, especially when the concentrations in the nutrient solution of this elemen were between 60 and 125 mg L^−1^. Summing up, according to our results, 10 of the measured traits were not affected by the treatments tested. Instead, 17 traits were enhanced, and only six were reduced, especially when the Si was tested at its highest level (250 mg L^−1^ Si). Therefore, most of the traits were positively affected by Si under our experimental conditions.

Silicon enters plant cells as an under-saturated solution of Si(OH)_4_ and yet it is found as amorphous hydrated silica within a plant. Biogenic silica cannot be formed spontaneously unless the concentration of Si(OH)_4_ in a plant exceeds solubility limits (Exley, 2015). Our treatments contributed with 60, 125 and 250 mg L^−1^ Si (in the form of CaSiO_3_) to the nutrient solution, respectively, which could result in differential accumulation rates within the plant cells, and thus triggered differential effects in a hormetic manner (either neutral, positive or negative) to the plant. Exogenous Si supply to nightshade plants including tomato (*Solanum lycopersicum* L.) (Gowda et al., 2015), eggplant (*Solanum melongena* L.) (Dasgan, Akhoundnejad & Çaglayangil, 2016) and pepper (Manivannan et al., 2016) has resulted in increased growth and productivity, both when crops are cultivated in the soil as well as in some soilless-cultivated greenhouse plants (Savvas & Ntatsi, 2015). In particular, chili pepper has exhibited an 8.4% yield increase in response to Si, as compared to the control (Liu et al., 2011).

In plants, Si has been proven to increase rigidity by strengthening cell walls and provide mechanical support to the canopy (Guerriero, Hausman & Legay, 2016; Yang et al., 2018). It has been also proven that the matrix polysaccharide (1;3,1;4)-β-D-glucan is involved in Si-dependent strengthening of cell walls (Kido et al., 2015). Silica deposition in the form of phytoliths (i.e., solid particles of polymerized SiO_2_) in cell walls may alter the anatomy of the plant tissues. Such changes in turn trigger some beneficial effects (Raven, 2001; Piperno et al., 2002; Ma, 2004; Savvas et al., 2007), while direct or indirect involvement of Si in plant metabolism may also occur (Epstein, 1999; Liang et al., 2003; Zhu et al., 2004). Indeed, in some plant species the absence of Si may cause structural weakening, a smaller size, lower development and viability, and greater susceptibility to environmental stressors. Conversely, the presence of Si avoids water loss through cuticular transpiration and increases the elasticity of the cell wall during plant growth by interacting with pectins and polyphenols, and gives greater general mechanical resistance (Wang et al., 2017). In the present study, plant height was greater with doses of 60 and 125 mg L^−1^ Si 7 dat, while after 21 days plant height increased in the treatments with the highest Si concentrations tested (i.e., 125 and 250 mg L^−1^). These results coincide with the greater height observed in rice plants receiving high doses of SiO_2_ (Cuong et al., 2017). However, in coffee (*Coffea arabica* L.), control plants showed greater height than seedlings supplied with 2 mmol L^−1^ Si (Cunha et al., 2012). This confirms that Si application may actually have different effects on different plant genotypes. Indeed, Si concentration in plants will be influenced primarily by the phylogenetic position of the plant rather than by the environmental conditions in which plants are established, such as soil parent materials and factors affecting Si adsorption-disorption processes (i.e., pH of the soil solution, water availability, temperature, and accompanied ions, among others) (Hodson et al., 2005; Yan et al., 2018; Artyszak, Gozdowski & Kucińska, 2019). Unlike other elements, Si is abundant in nearly all soils, so environmental criteria do not significantly impact Si accumulation in plants but rather their intrinsic mechanisms to absorb and transport Si (Ma & Takahashi, 2002), which is mediated by Si channels or transporters. Even though Si accumulation is a phylogenetic feature, the availability of Si in the soil may influence, at least partially, the amount of Si absorbed by plants (Guntzer, Keller & Meunier, 2012). Under our experimental conditions, with the addition of Si, root length decreased significantly at 7, 14 and 21 dat, while at 28 days the application of 125 and 250 mg L^−1^ Si resulted in root length means similar to the control. In carnation (*Dianthus caryophyllus* L.), the greatest values in stem length and root length with and without saline stress were obtained with the application of 50 mg L^−1^ Si; when doubling the dose, the effect was similar to the control (Soundararajan et al., 2015). In chili pepper, increasing Si levels (0, 50, 75 and 100 mg L^−1^) had no significant effects on stem and root length (Jayawardana, Weerahewa & Saparamadu, 2015). The root system displays considerable plasticity in its morphology and physiology in response to variability within its environment (Ortíz-Castro et al., 2009). Decreased root length can be beneficial for plants because they can target their energy and metabolism to increase the shoot system instead of the root system. A less developed root system may indicate that water and nutrients are available nearby. In some cases, especially in nutrient deficient environments, plants have to explore more extensive areas and thus develop a more expanded root system to reach water and nutrients, to the detriment of the shoot system. Hence, our results can be interpreted as beneficial.

Under our experimental conditions, number of leaves, root volume, number of flower buds per plant, as well as fresh and dry biomass weight of flowers, leaves and roots were statistically similar among treatments tested. Interestingly, flower buds were bigger in the treatments with Si. Despite the absence of significant effects of Si on the number of leaves, leaves developed more area in the treatment with 125 mg L^−1^ Si. Similarly, the application of increasing doses of Si (1 and 2 mM Si) in cherry tomato did not affect root volume and stem diameter (Haghighi & Pessarakli, 2013). Nevertheless, in sugar beet (*Beta vulgaris* L. subsp. *vulgaris*) foliar applications of Si increased root yield by 7.5–25.1%, biological sugar yield by 7.1–23.2%, and commercial yield of sugar by 4.8–22.2%, compared to the control treatment (without Si application) (Artyszak, 2018). Coincidently, pepper plants treated with 1.5 mM Si (supplied as K_2_SiO_3_) for 15 days increased shoot length, shoot diameter, root length, number of roots and fresh biomass weight in comparison to control plants (Manivannan et al., 2016). Nonetheless, in coffee seedlings, the number of leaves and internodes showed no statistical difference among treatments (Cunha et al., 2012). Thus, the application of Si can differentially affect plant growth and development, depending on the internal mechanisms that the genotype has to metabolize this element (Ouellette et al., 2017). Biological responses of plants to Si can be a consequence of the apoplastic obstruction caused by excesive Si deposition in the cell wall (Coskun et al., 2019), which can trigger hormetic effects (Mburu et al., 2016; Agathokleous & Calabrese, 2020). In plants, Si is deposited as the solid, hydrated oxide SiO_2_·nH_2_O, known as silica gel, following polymerization of orthosilicic acid (Si(OH)_4_ or H_4_SiO_4_; the only form of Si available to plants) (Gropper & Smith, 2018). Polymerization of orthosilicic acid into silica gel can result in stimulation or inhibition of plant responses, depending on the severity it reaches (Ma, Miyake & Takahashi, 2001; Exley, 2015; Montpetit et al., 2012).

Fresh and dry biomass weights of leaves and roots were not affected by Si treatments under our experimental conditions. Conversely, in stems these variables were enhanced by Si (Figs. 4A and 4B). Similarly, the application of Si in wheat increased the dry biomass of stems by 19.6%, 23.8%, 36.5% and 32.6% with 24, 50, 100 and 200 mg L^−1^ Si respectively, compared to the control (0 mg L^−1^ Si), while applying 400 and 800 mg L^−1^ Si reduced this variable to a lower level than the control (Mali & Aery, 2008).

In two maize (*Zea mays* L.) cultivars grown under normal conditions (i.e., no stress applied), the application of Si resulted in slight fresh biomass increases, possibly due to the improvement of the photosynthetic apparatus and increasing water use efficiency, though no differences were observed compared to the control (Khan et al., 2017). In wheat, the application of 1–10 g Si per plant increased the aerial biomass, but as the dose increased, the biomass decreased (Neu, Schaller & Dudel, 2017). In cotton (*Gossypium hirsutum* L.), wheat, and canola (*Brassica napus* L.), the application of 1.5 mmol L^−1^ Si increased dry weight by 8%, 30% and 30% and fresh weight by 10%, 33% and 16%, respectively (Mehrabanjoubani et al., 2015). Moreover, the pre-treatment of maize kernels with 1.5 mM Si significantly increased dry and fresh weight and leaf area of plants (Abdel Latef & Tran, 2016). In two cucumber (*Cucumis sativus* L.) cultivars established in conventional and saline soils, Si applications increased the dry biomass of the aerial part and roots, which was related to a higher activity of antioxidant enzymes (Khoshgoftarmanesh, Khodarahmi & Haghighi, 2014). In aloe [*Aloe vera* (L.) Burm. f.] plants grown under normal and saline conditions, the fresh weight of the leaves increased with the addition of Si, though significant differences with respect to the control were only evident when plants were exposed to salt stress, which was associated with a higher concentration of K^+^ in leaves, stems, and roots, and a lower concentration of Na^+^, due to the stabilization of the activity of a proton pump (Xu, Ma & Liu, 2015). Moreover, the application of K_2_SiO_3_ caused a higher Si concentration in better developed tissues of carnation plants in vitro, compared against the application of CaSiO_3_ (Manivannan et al., 2017), which proves that the Si source used also influences the response observed in the plant. Si may affect plant growth by regulating the levels of endogenous phytohormone and conferring resistance to the turgor pressure ocuring during cell elongation (Luyckx et al., 2017b). Indeed, in Si-treated rice third leaves, the epidermal cell length increased, especially in the basal regions, without any effect on the number of cells, showing that Si promoted cell elongation but not cell division. Si also increased the cell wall extensibility significantly in the basal regions of rice third leaves, which indicates that Si stimulates growth of plant leaves by increasing cell wall extensibility (Hossain et al., 2002).

The role of chlorophylls in photosynthesis is vital, and Si has been demonstrated to enhance both chlorophyl biosynthesis and photosynthetic activity in various plant species. Important deposition of Si has been found in leaves, which results in greater tissue rigidity and more erect leaf blades. These conditions favor light interception, stimulate greater CO_2_ absorption, and decrease excessive transpiration, which consequently results in higher photosynthetic rates and increased yields (Detmann et al., 2012; Savvas & Ntatsi, 2015).

In carnation, the application of Si increased the activity of PsaA and PsbA enzymes, which stimulated the efficiency of the photosystem II and the electron transference speed (Manivannan et al., 2017). In rice, Si stimulated photosynthetic indicators and the expression of genes related to photosynthesis, like *PsbY*, *cffv*, *PetC* and *PetH* (Song et al., 2014). In the Japanese honeysuckle (*Lonicera japonica* Thunb.), the application of Si helped to maintain the ultrastructure of the chloroplast (Gengmao et al., 2015). In our research, the highest *a*, *b*, and total chlorophyl values in leaves and stems were observed with the application of 125 mg L^−1^ Si, though 250 mg L^−1^ Si and 60 mg L^−1^ Si did not increase the concentrations of these molecules. In cacao (*Theobroma cacao* L.), the addition of 1.5 mg mL^−1^ SiO_2_ increased the photosynthetic rate and mitigated oxidative stress (Zanetti et al., 2016). In wheat, applications of 150 mg L^−1^ Si to the soil significantly increased the concentration of chlorophylls, while the application of 50 and 100 mg L^−1^ Si had no significant effect, compared to the control (Saleh, Najafi & Oustan, 2017). Low Si doses (one mM) increased the concentration of chlorophylls *a* and *b* in hydroponically grown wheat, compared to the control, but when the Si level increased to four mM, the chlorophyl concentration decreased (Hajiboland, Cherghvareh & Dashtebani, 2017). Also, the application of 150 mg L^−1^ Si in maize established in an alluvial soil increased the concentration of total chlorophylls and the photosynthetic rate, compared to the control (Xie et al., 2014). A more specific study on maize determined that applying two mM Si stimulated the concentration of chlorophylls *a*, *b*, and total by 22%, 43% and 26%, respectively, as compared to the control (Barbosa et al., 2015). This has also been observed in wheat exposed to drought stress (Maghsoudi, Emam & Ashraf, 2015). A significant increase in the concentrations of chlorophylls *a* and *b* in pepper cv. Giant Vermelho and a concomitant stimulation of the activity of the photosynthetic apparatus combined with the architecture of the plant were promoted by the application of Si (Pereira et al., 2015). The addition of 0.25, 1.00 and 1.75 µmol Si to tomato cv. Super Marmante and Santa Cruz exposed to water deficit increased the levels of chlorophylls *a*, *b*, and total, which were related to a more efficient protection of the photosynthetic apparatus (Silva et al., 2012). In maize grown on alkaline soils, the application of 1.5 mM Si significantly increased the concentration of photosynthetic pigments and decreased the negative impact of stress (Abdel Latef & Tran, 2016). Also, the application of three mM Si from SiO_2_ to rice plants significantly increased the chlorophyl *a*/*b* ratio (Ramírez-Olvera et al., 2019). In our study, the chlorophyl *a*/*b* ratios in leaves and stems were higher with the 250 mg L^−1^ Si treatment, while the lowest chlorophyl *b* in stem was similar to that of the control, with no significant differences among the rest of the treatments. Importantly, under drought stress, Si decreased the decomposition of chlorophylls (Ma et al., 2004), while a Si-related increase of the photosynthetic capacity in bent-grass (*Agrostis palustris* Huds.) was associated with enhanced chlorophyl content (Schmidt, Zhang & Chalmers, 1999). Furthermore, supplying Si to salt-stressed wheat plants can restore the chlorophyl level to that of non-stressed plants (Tuna et al., 2008). In potato (*Solanum tuberosum* L.), a significant increase of net photosynthetic rates after both soil and foliar application of Si to non-stressed plants was observed, which was associated with a significant increase in the concentrations of chlorophyl *a* and carotenoids (Pilon, Soratto & Moreno, 2013).

The highest concentration of free amino acids in leaves was found in pepper plants treated with 60 mg L^−1^ Si. Applying 125 mg L^−1^ Si resulted in amino acid concentrations similar to the control, while in plants treated with 250 mg L^−1^ Si the concentration of amino acids decreased significantly. In stems, plants treated with 60 and 125 mg L^−1^ displayed similar free amino acid concentrations to the control, while at 250 mg 125 mg L^−1^ stems exhibited lower concentrations of these molecules as compared to the other three treatments. In roots, all Si treatments tested exhibited higher concentrations of amino acids as compared to the control. The application of 0.25, 1.00 and 1.75 µM Si in the pepper cultivars Ikeda and Giant Vermelho exposed to water stress increased the concentrations of soluble amino acids, although significant differences with respect to the control were only observed in the Ikeda cultivar treated with 0.25 µM Si (Pereira et al., 2013). At the biochemical level, Si has been shown to improve antioxidant capacity and photosynthetic activity (Manivannan et al., 2016), and also contributes to the osmotic adjustment by increasing the synthesis of amino acids such as proline (Rezende et al., 2017) and other essential amino acids (Johnson et al., 2017). In maize seedlings under normal conditions, Si did not affect the content of free amino acids, but when the plants were exposed to alkaline stress, the amino acids significantly increased, and this increase was greater with the addition of Si (Abdel Latef & Tran, 2016). Strawberry plants treated with Si did not increase their concentration of amino acids in leaves and roots, but protein concentration did increase, which proves that this element stimulates protein synthesis and therefore plant development (Ouellette et al., 2017). In the rice cultivars Shengdao 14 and Huaidao 11, Si applications increased the concentrations of Asp, Glu, Ser, Ala, Tyr, Arg and Pro by 12%, 3.55%, 9.15%, 5.06%, 28.77%, 13.24% and 10.83%, respectively, and Thr, Ile, and Leu by 11.50%, 8.82% and 4.75%, respectively, compared to the control. Grain yield and protein concentration also increased as compared to the control (Liu, Zhou & Sun, 2017). Likewise, in maize plants exposed to alkaline stress, the highest content of free amino acids was observed in plants treated with 25 mM Na_2_CO_3_ and 1.5 mM Si (Abdel Latef & Tran, 2016). Therefore, Si has an impact on amino acid concentrations in different plant species.

The concentration of total soluble sugars was higher in treatments with 125 mg L^−1^ Si in leaves, stems and roots. Similarly, Si-treated tomato plants had higher concentrations of sugars and improved yields (Jarosz, 2014). Moreover, the application of 1.5 mM Si mitigated the effects caused by alkaline stress, increasing the accumulation of soluble sugars (Abdel Latef & Tran, 2016). In the present study, the increase of total soluble sugars observed in plants treated with 125 mg L^−1^ Si was associated with the increase in chlorophyl *a* and total chlorophyl observed in plants under the same treatment (Table 2). The chlorophyl increase triggered by Si favors light absorption through the leaves, thus increasing the photosynthetic activity and the content of soluble sugars (Savvas & Ntatsi, 2015; Sakurai et al., 2017). However, there can be different responses between genotypes, as observed in the Giant Vermelho pepper variety, where Si increased the concentrations of soluble sugars while in the Ikeda variety the concentration of sugars decreased (Pereira et al., 2015). Similarly, the application of Si decreased the levels of total soluble sugars in tomato exposed to water deficit (Silva et al., 2012). Sugars are important components of plant cell walls. It is plausible that the complexation of Si with cell wall macromolecules takes place via the stabilization of sugars, in a manner analogous to the borate-mediated formose reaction (He et al., 2013; Guerriero, Hausman & Legay, 2016).

Our results are in full agreement with those reported by Manivannan et al. (2016), who found increased growth in unstressed pepper plants treated with 1.5 mM Si (supplied as K_2_SiO_3_) for 15 days in comparison to untreated plants (control). Under control conditions (i.e., in the absence of stress conditions), Si probably activates the metabolic status of the plant by making it more efficient in response to external stimuli (Luyckx et al., 2017a). For instance, in rice plants under unstressed conditions, Si causes alteration of the C/N balance in the source-sink relationship during grain development, thus increasing grain yield, which, in turn, exerts a feed-forward stimulation of photosynthetic rates via enhanced mesophyll conductance and alters primary metabolism (Detmann et al., 2012; Detmann et al., 2013).

Apart from improving plant performance under unstressed conditions, Si has been shown to play an important role in alleviating damage caused by both biotic and abiotic stresses (Manivannan & Ahn, 2017). For instance, improved plant defense against arthropods under Si supplementation has been associated with a mechanical form of protection (Reynolds, Keeping & Meyer, 2009; Reynolds et al., 2016). Si acts as a physical defense, increasing the abrasiveness of the leaves and leading to the increased wear of mandibles chewing herbivores (Kvedaras & Keeping, 2007), thus reducing palatability and digestibility of plants for herbivores (Massey & Hartley, 2009). Importantly, abrasiveness of plant tissues is more influenced by phytolith morphology than by Si concentration applied (Hartley et al., 2015). Moreover, physical strength of the leaf resulting from Si accumulation may induce mechanical protection and thus lower the rate of infection of some pathogens (Zhang et al., 2013; Schurt et al., 2014; Ning et al., 2014). Priming of plant defense responses, alterations in phytohormone homeostasis, and networking by defense signaling components are all potential mechanisms involved in Si-triggered resistance responses (Wang et al., 2017).

As sessile organisms, plants have evolved unique mechanisms enabling them to face the complexity of environmental changes, developing vital strategies to reach optimal growth, development and reproduction. These mechanisms include signal perception and transduction processes, so that plants may construct a response to an environmental signal. Indeed, even under unstressed conditions, Si might act as a signal to promote amino acid remobilization to support the increased N demand during grain development in rice (Detmann et al., 2012; Detmann et al., 2013), suggesting that Si may in fact have signaling roles in plants. Since Si interacts with key components of plant signaling systems, including its binding to the hydroxyl groups of proteins involved in cell signaling and its interaction with cationic co-factors of enzymes influencing stress responses, it can act as a signaling modulator in a manner similar to a second messenger (Fauteux et al., 2005; Luyckx et al., 2017a). Therefore, future recommendations to agronomists will include Si applications to fields that are deficient in the element (Liang et al., 2015; Coskun et al., 2019), in particular with a view to the rapid pace of global climate change and the increased incidence of inclement and extreme weather events (Lobell, Schlenker & Costa-Roberts, 2011; Cai et al., 2014; Myers et al., 2014) negatively affecting crop productivity (Cottrell et al., 2019; Raza et al., 2019). Such environmental alterations lead to new challenges for agriculture and food production. Si may thus be of paramount importance for triggering adaptive responses of plants in harsher environments, but the precise molecular cues involved in these processes still need to be clearly identified.

The application of Si has resulted in the enhancement of quantitative and qualitative traits in different crop species not only under unstressed but also under stressed environments (Manivannan & Ahn, 2017). It has been proved that Si may regulate several physiological, biochemical, and antioxidant responses in plants to combat abiotic and biotic stresses. For instance, Si and Fe differently alleviate Cu toxicity in cucumber. In particular, Si-mediated alleviation of Cu toxicity was directed toward Cu tolerance while Fe-alleviative effect was due to a dramatic decrease in Cu accumulation (Bosnić et al., 2019). Goethite-modified biochar can combine the beneficial effects of biochar and Fe for remediation of Cd- contaminated soil, while improving key physiological and biochemical attributes of rice plants (Kashif-Irshad et al., 2020). Furthermore, P application helps decrease Cd concentrations in wheat shoots and increase gas exchange attributes and antioxidant enzymes (Arshad et al., 2016), which can be implemented in a general scheme aimed at controlling Cd concentrations in other plant species. Interestingly, foliar application of ascorbic acid also alleviated the detrimental effects of drought stress in maize plants by improving their antioxidative defense system (Noman et al., 2015). Since Si can also attenuate the toxic effects of heavy metals such as Cd (Lu et al., 2018; Wu et al., 2018) as well as drought and salt stress (Rizwan et al., 2015) in plants, these approaches using biochar, P and ascorbic acid could also be employed to mitigate the detrimental effects of these and other stresses in plants in combination with Si.

## Conclusions

Silicon supplementation to pepper plants during the early developmental stage resulted in hormetic-like biphasic dose–responses, with stimulatory effects at low–doses and inhibitory responses at high–doses. Beneficial effects were evident in numerous variables such as leaf area, plant height, fresh and dry biomass weight of stems, total free amino acids in leaves and roots, total soluble sugars in leaves and stems, and chlorophyl concentrations, especially in plants treated with 60 and 125 mg L^−1^ Si. However, some negative effects were observed at the highest concentration applied (i.e., 250 mg L^−1^ Si), especially on root length, chlorophylls concentrations, stem diameter, and total free aminoacids in leaves and stems. Therefore, pepper is a good candidate crop to benefit from Si, though further research is required to define the optimal doses and stages to apply it among different pepper genotypes.

## Supplemental Information

10.7717/peerj.9224/supp-1 Supplemental Information 1 Growth parameters of pepper (*Capsicum annuum* L.) plants treated with different doses of silicon (0, 60, 125 and 250 mg L^−1^ Si) under greenhouse conditions in hydroponics.

10.7717/peerj.9224/supp-2 Supplemental Information 2 Concentration of chlorophylls (Chl) in pepper (*Capsicum annuum* L.) plants treated with different doses of silicon (0, 60, 125 and 250 mg L^−1^ Si) under greenhouse conditions in hydroponics.

10.7717/peerj.9224/supp-3 Supplemental Information 3 Concentration of amino acids (AA) in pepper (*Capsicum annuum* L.) plants treated with different doses of silicon (0, 60, 125 and 250 mg L^−1^ Si) under greenhouse conditions in hydroponics.

10.7717/peerj.9224/supp-4 Supplemental Information 4 Concentration of total soluble sugars (SS) in pepper (*Capsicum annuum* L.) plants treated with different doses of silicon (0, 60, 125 and 250 mg L^−1^ Si) under greenhouse conditions in hydroponics.

## Additional Information and Declarations

### Data Availability

The following information was supplied regarding data availability: The raw measurements are available in the Supplemental Files: Data S1–Data S4.
